# Salvage Surgery for Epidermal Growth Factor Receptor-Mutant Lung Cancer With Osimertinib Resistance: A Case Report

**DOI:** 10.7759/cureus.78461

**Published:** 2025-02-03

**Authors:** Shinya Otsuka, Yutaro Nagano, Makoto Shioya, Hiroya Ohkawa, Tatsuya Kato

**Affiliations:** 1 Department of Thoracic Surgery, Hokkaido University Hospital, Sapporo, JPN; 2 Department of Respiratory Medicine, Otaru General Hospital, Otaru, JPN; 3 Department of Surgical Pathology, Hokkaido University Hospital, Sapporo, JPN; 4 Dapartment of Thoracic Sugery, Hokkaido University, Sapporo, JPN

**Keywords:** drug resistance, egfr t790m mutation, lung cancer, molecular targeted therapies, osimertinib, salvage surgery

## Abstract

Recent advancements in lung cancer treatment, including molecular targeted therapies and immune checkpoint inhibitors, have significantly improved patient outcomes and extended survival. However, the efficacy and safety of salvage surgery for lesions resistant to targeted therapy like osimertinib remain poorly understood. This report highlights a case of salvage surgery performed on a patient with epidermal growth factor receptor (*EGFR*)-mutant lung cancer resistant to osimertinib. A 60-year-old woman was diagnosed with lung adenocarcinoma (cT1cN3M0, cStage IIIB) harboring an *EGFR* exon 19 deletion (L747-A750>P). Initial treatment with afatinib yielded a favorable tumor response for 18 months, but disease progression ensued. Osimertinib was subsequently initiated after detection of the *EGFR* T790M mutation, demonstrating initial efficacy. Nineteen months later, regrowth of a lung lesion was observed, although radiographic evaluation showed no lymph node enlargement, with disease confined to the right upper lobe. A robot-assisted right upper lobectomy with lymph node dissection was successfully performed. During surgery, the patient experienced refractory atrial fibrillation, which was successfully managed with defibrillation. Pathological analysis confirmed metastasis to the right upper mediastinal lymph nodes. The *EGFR* exon 19 deletion mutation was detected in the resected tumor, while the T790M mutation was absent. The patient has remained disease-free for 10 months without additional treatment. Salvage surgery following molecular targeted therapy for lung cancer has been reported to be effective for selected patients. However, the mechanisms of resistance to osimertinib are diverse, and the efficacy of salvage surgery for patients with osimertinib resistance is not well understood. In this case, although the postoperative observation period is still short, salvage lung resection suggests the possibility of leading to favorable disease control. With a thorough evaluation of surgical indications and appropriate management of several complications associated with the prior treatment, salvage surgery can be a valuable and viable option for selected patients.

## Introduction

In recent years, pharmacological advancements in lung cancer treatment have progressed rapidly. Over the past decade, the introduction of numerous molecular targeted therapies and immune checkpoint inhibitors into clinical practice has markedly improved the prognosis for patients with advanced lung cancer. In addition, the use of these therapies in perioperative settings has also developed. Regarding perioperative molecular targeted therapy, it has been reported that administering osimertinib postoperatively in patients with epidermal growth factor receptor (*EGFR*) mutations, or alectinib in patients with anaplastic lymphoma kinase (*ALK*) mutations, extended relapse-free survival (RFS) following surgery [1,2]. Despite these advances, the efficacy and safety of salvage surgery for progressive or residual lesions following molecular targeted therapy, particularly after treatment with osimertinib, remain insufficiently studied. Osimertinib targets *EGFR* T790M mutation and irreversibly binds to cysteine-797. It can contribute to better disease control in advanced lung cancer with *EGFR* mutations compared to existing *EGFR*-tyrosine kinase inhibitors (*EGFR*-TKIs) [3,4]. However, with long-term use, it acquires drug resistance through mechanisms dependent on *EGFR* mutations, alternative pathway activation, or histological transformation [5]. As treatments after resistance acquisition, combinations of platinum chemotherapy and immunotherapy, as well as antibody drugs, are being developed [6,7]. However, real-world usage experience has not accumulated, and the treatment is often challenging. This report presents a case of salvage surgery performed on a patient with *EGFR*-mutant lung cancer who developed resistance to osimertinib.

## Case presentation

**Figure 1:**
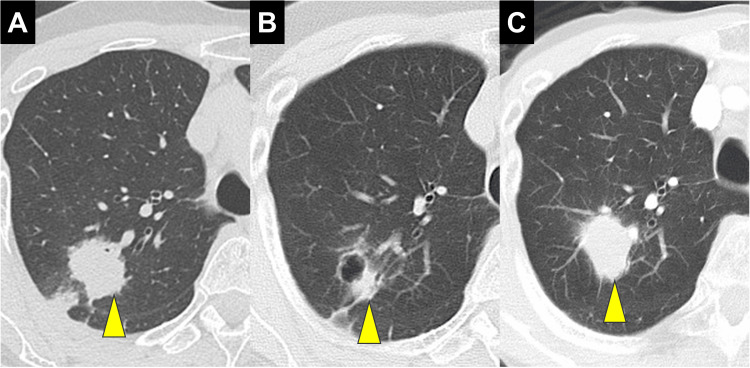
Progression of CT findings during molecular targeted therapy The target nodule is emphasized with arrowheads. (A) A CT scan prior to drug treatment revealed a 27 mm nodule in the right upper lobe. (B) The lung nodule reduced to 12 mm in size after 13 months of afatinib treatment. (C) A subsequent CT scan showed re-progression of the lung lesion to 30 mm 19 months after starting osimertinib.

**Figure 2:**
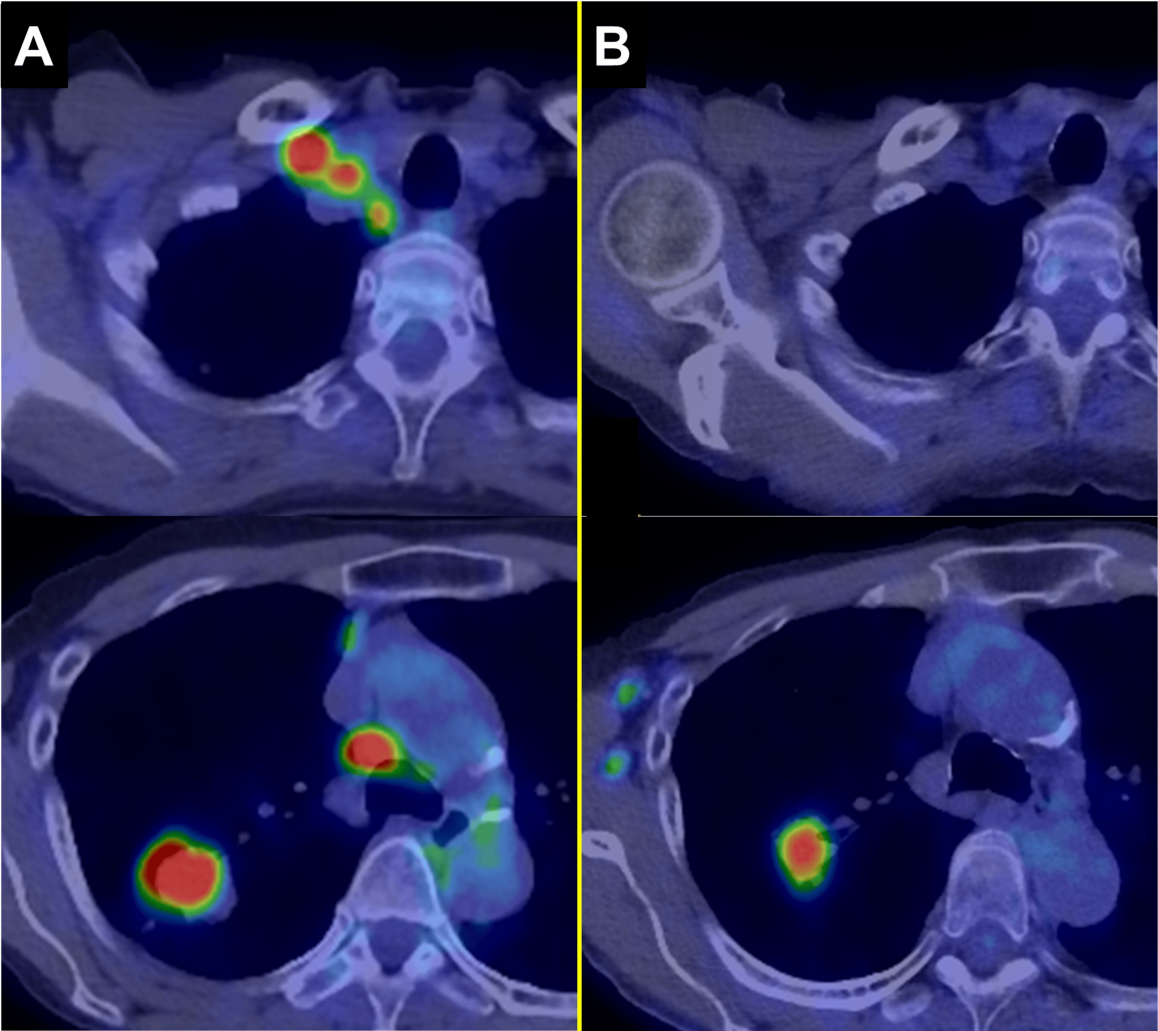
PET-CT findings before and after molecular targeted therapy (A) At the initial visit, PET-CT demonstrated FDG uptake with an SUVmax of 12 in the primary tumor and SUVmax values ranging from 8 to 13 in the right supraclavicular and mediastinal lymph nodes. (B) After targeted therapy (one month before the salvage surgery), FDG uptake with an SUVmax of 16 was observed in the primary tumor, while no uptake was detected in the lymph nodes. Therefore, it was decided to proceed with salvage surgery.

A 60-year-old woman presented with an abnormal chest shadow on imaging in August 2018. The patient had a history of paroxysmal atrial fibrillation (Af) and was being treated with bisoprolol. Computed tomography (CT) scans identified a nodule in the right upper lobe (Figure 1A), and positron emission tomography (PET)-CT revealed fluorodeoxyglucose (FDG) uptake in the right supraclavicular and mediastinal lymph nodes (Figure 2A).

A bronchoscopic examination confirmed lung adenocarcinoma, staged as cT1cN3M0 cStage IIIB. Genetic testing identified an *EGFR* exon 19 deletion (L747-A750>P) and a low PD-L1 tumor proportion score (TPS, 0-10%). Radical resection and definitive chemoradiotherapy were deemed unfeasible at the time due to multiple lymph node metastases, and afatinib-targeted therapy was initiated.

Afatinib 20 mg led to significant reductions in the lung lesion and lymph node metastases (Figure 1B). However, after 18 months, the lung tumor began to enlarge. A liquid biopsy at that time did not detect the *EGFR* T790M mutation. Three years and seven months after initiating afatinib, a repeat bronchoscopy confirmed the presence of the T790M mutation, and osimertinib therapy was started. Osimertinib 80 mg was initially effective, but after 19 months, regrowth of the lung lesion was observed. Radiographic examinations revealed no lymph node enlargement, with disease confined to the right upper lobe, prompting the decision to proceed with salvage surgery (Figures 1C, 2B). Mild liver dysfunction (grade 1 according to the Common Terminology Criteria for Adverse Events [8]) associated with treatment with osimertinib was observed; however, no other significant complications were noted during targeted therapy.

The preoperative electrocardiogram showed no signs of arrhythmia. Robot-assisted right upper lobectomy with upper mediastinal lymph node dissection (ND2a-1) was performed. The procedure was facilitated by the absence of pleural adhesions and minimal fibrosis or calcified lymph node adhesion, allowing safe dissection of blood vessels and the bronchus. During surgery, the patient experienced hypotension due to paroxysmal Af, which was successfully managed with defibrillation. The operation was completed uneventfully, with a total duration of two hours and 28 minutes and a blood loss of less than 50 mL.

**Figure 3:**
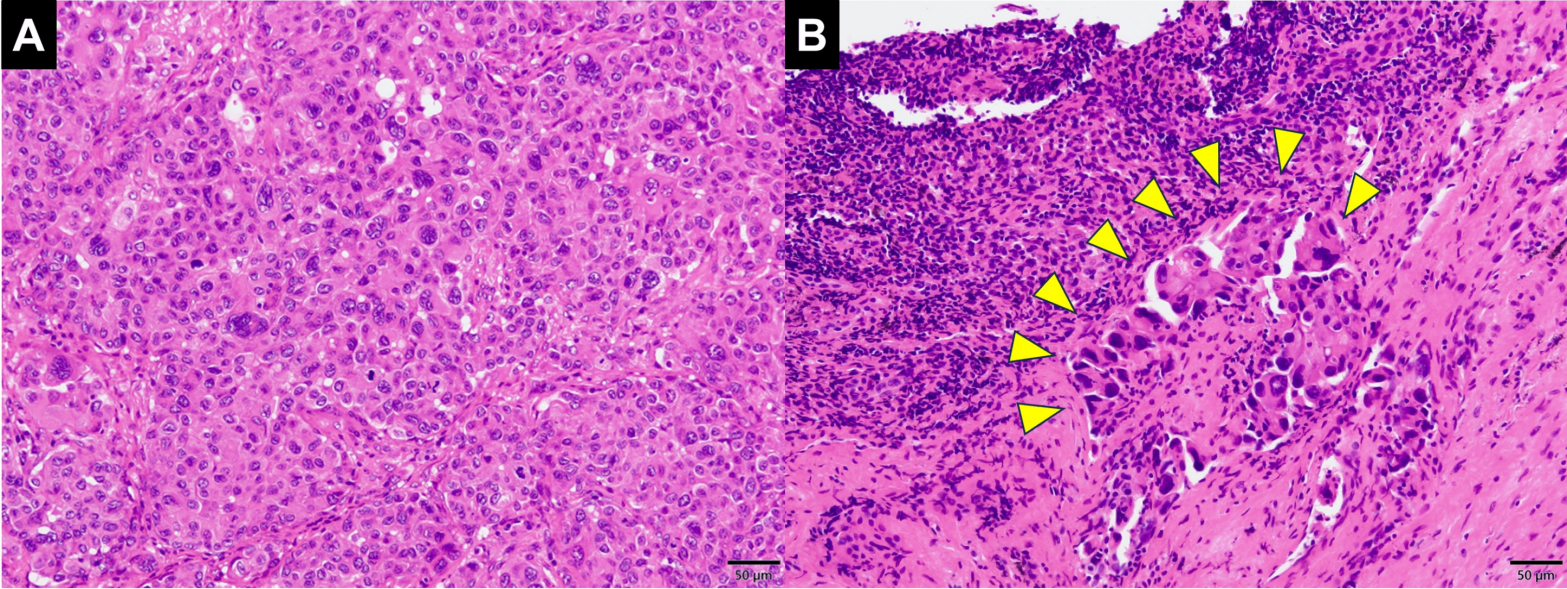
Pathological findings of the resected lung tumor and mediastinal lymph node (A) Tumor cells with enlarged nuclei were proliferating, forming cohesive glandular structures and solid cell nests in the primary lung lesion. (Hematoxylin-Eosin [HE] staining; scale bar: 50 μm). (B) Atypical cells were identified in the upper mediastinal lymph node (arrowhead). (HE staining; scale bar: 50 μm)

Postoperative recovery was smooth, and the patient was discharged seven days after surgery. Pathological examination revealed metastasis to the right upper mediastinal lymph nodes (Figure 3).

The *EGFR* exon 19 deletion was detected in the resected tumor, while the T790M mutation was absent. The patient has remained alive and disease-free for 10 months without further treatment or signs of recurrence.

## Discussion

To our knowledge, only three reports have examined cases of salvage surgery following molecular-targeted therapy for lung cancer [9-11]. These studies primarily involve patients treated with afatinib or gefitinib for *EGFR* mutations or alectinib for *ALK* mutations, with limited data available on post-osimertinib surgical treatment. In these reports, the three-year RFS rates were relatively low (20-30%), but overall survival (OS) reached approximately five years - considered favorable for initially unresectable lung cancer. Notably, Chen et al. found that patients undergoing salvage surgery after disease progression following molecular targeted therapy experienced worse postoperative OS and RFS compared to those with stable disease. Furthermore, long-term prognosis improvements appear unlikely for patients with pleural dissemination or distant metastases, emphasizing the need for careful patient selection [9].

Salvage surgery serves two main purposes. First, it enables detailed genetic and pathological evaluations of residual tumors, aiding in the development of appropriate treatment strategies. Radiographic assessments may suggest significant residual tumors, which could primarily consist of less invasive forms, such as adenocarcinoma in situ or necrotic tissue. Conversely, even when lesions appear reduced or absent following molecular targeted therapy, viable tumor cells may persist microscopically. A phase II trial evaluating osimertinib in the neoadjuvant setting indicated that achieving a major pathological response may be challenging with targeted therapy alone [12].

Second, surgical resection reduces local tumor burden and tumor heterogeneity, helping to prevent the emergence of new genetic mutations and drug resistance. Tumor cells with acquired resistance may exist before clinical disease progression becomes evident [11]. Early resection of potentially drug-tolerant cells can enhance postoperative disease control and OS.

This case involved an uncommon L747-A750>P mutation, a rare variant accounting for only 4% of all exon 19 deletions. This mutation is potentially associated with a poorer prognosis but exhibits higher sensitivity to afatinib than osimertinib, justifying the use of afatinib as first-line treatment [13]. Besides the L747-A750>P mutation, numerous rare genetic mutations in *EGFR* have been reported. It is not well understood which is more effective, osimertinib or older generation *EGFR*-TKIs like afatinib for those cases [14]. Of course, the efficacy of salvage surgery is also unknown, but it could be a feasible option when drug treatment becomes challenging.

Genetic analysis of the resected specimen revealed the absence of the T790M mutation, a key factor in osimertinib resistance. Resistance mechanisms in such cases are complex, with T790M loss often linked to *EGFR*-independent pathways, such as mesenchymal-epithelial transition factor (*MET*) amplification, phosphatidylinositol-4,5-bisphosphate 3-kinase catalytic subunit alpha (*PIK3CA*), Kirsten rat sarcoma viral oncogene homolog (*KRAS*), or human epidermal growth factor receptor 2 (*HER2*) mutations. These *EGFR*-independent mechanisms typically result in shorter treatable durations and worse prognoses compared to *EGFR*-dependent resistance, such as the C797S mutation [5,15]. In such cases, it has been reported that even though the *EGFR* exon 19 deletion is positive and the T790M mutation is absent, sensitivity to first-generation *EGFR*-TKIs is not restored [5]. In our case, as resistance to osimertinib had already developed preoperatively, and the efficacy beyond progressive disease was not anticipated due to L747-A750>P mutation, postoperative targeted therapy was not administered.

The perioperative risks associated with molecular targeted therapy remain poorly defined. A neoadjuvant osimertinib study reported a 25% incidence of postoperative Af [12]. In the present case, the patient experienced paroxysmal Af prior to lung cancer development, and refractory Af was observed during surgery. Though it remains unclear whether this was linked to targeted therapy, especially in patients with a history of Af, careful attention must be paid to refractory Af during and after surgery.

In addition, fibrocalcified lymph nodes and tumor necrosis can complicate the dissection of blood vessels and bronchi. Chen et al. reported intraoperative bleeding in two cases due to lymph node degeneration during salvage lung surgery, although its association with preoperative treatment is uncertain [9]. In the present case, there was no evidence of calcified lymph nodes or significant fibrosis; however, maintaining vigilance for potential tissue degeneration during surgery is crucial.

In this case, surgery was performed due to re-progression following targeted therapy, but no recurrence has been observed to date postoperatively. During molecular targeted therapy, no distant metastasis was observed consistently, and only lymphatic metastasis was present, which may be associated with a favorable postoperative prognosis.

Despite the genetic complexity in this case, including factors complicating treatment strategies, salvage surgery achieved effective disease control and suggested the coexistence of multiple resistance mechanisms, including *EGFR*-independent bypass activation. These findings can inform tailored treatment decisions following surgery. If recurrence occurs in the future, further surgical or drug treatment will be considered based on the patient's general condition, tumor growth rate, and genetic information to achieve long-term disease control. Advancements in genetic and molecular pathological analyses of lung cancer may enhance the role of salvage surgery, providing a bridge to subsequent therapies and improving overall patient outcomes.

## Conclusions

In this case, although the postoperative observation period is still short, salvage lung resection after resistance acquisition suggests the possibility of leading to favorable disease control. This approach can help guide subsequent treatment strategies and delay the emergence of new resistance mechanisms. With a thorough evaluation of surgical indications and appropriate management of several complications associated with the prior treatment, salvage surgery can be a valuable and viable option for selected patients.
